# Morphological and Chemical Characterization of a Novel Wild Tea Plant Resource with Naturally Low Caffeine and High Theobromine from Guangxi Province, China

**DOI:** 10.3390/plants15111642

**Published:** 2026-05-27

**Authors:** Qianting Ma, Zhongjun Yan, Xiaolu Yang, Aixiang Hou, Zhen Liu, Shuang Gan, Yihuan Yang, Yaojin Chen, Ruijin Qiu, Wenliang Wu

**Affiliations:** 1 Longping Agricultural College, Hunan University, Changsha 410082, China; mqt0303@163.com; 2 Key Laboratory of Liupao Tea Biology and Resource Utilization, Wuzhou Institute of Agriculture Sciences, Wuzhou 543003, China; ganshuang1995@163.com (S.G.); tyyhdyx@126.com (Y.Y.); qiongin@163.com (Y.C.); 3 Hunan Institute of Tea Research, Hunan Academy of Agricultural Sciences, Changsha 410125, China; yzj771104@163.com (Z.Y.); hnteascience@126.com (Z.L.); 4 College of Food Science and Technology, Hunan Agricultural University, Changsha 410128, China; xljc852995@stu.hunau.edu.cn (X.Y.); aixianghou@163.com (A.H.)

**Keywords:** wild tea germplasm, low caffeine, metabolomics, morphological characterization

## Abstract

Zhuyecha (ZYC, *Camellia* sp.) is a newly discovered special wild tea plant resource in Cangwu County, Wuzhou City, Guangxi Province, China. However, there have been few scientific studies on it. This study investigated its morphological and chemical characteristics. The results revealed that ZYC is an arbor-type, upright plant with lanceolate leaves. The flower has five petals, five sepals, a trifid style, and a glabrous ovary. ZYC exhibited a unique chemical profile distinguished from common tea cultivars. Compared with the control cultivated varieties (Hongyan 12, Fuyun 6, and Yinghong 9), ZYC fresh leaves contained significantly higher contents of theobromine, theophylline, and gallic acid, but considerably lower caffeine and lower total catechins. The principal catechins were *epi*-form/galloylated catechins EGCG and ECG. Notably, theobromine was the most abundant alkaloid in ZYC, followed by caffeine and theophylline. In ZYC fresh leaves, 15 differential non-volatile metabolites were identified, and 22 key aroma compounds were screened, including linalool, (*E*)-2-hexenal, hexanal, heptan-2-ol, and *β*-myrcene, among others. The volatile components were primarily alcohols, aldehydes, and esters, contributing to floral, fruity, and green aroma attributes. This work enhances the understanding of the botanical and phytochemical properties of ZYC and provides scientific guidance for further research and efficient utilization of this natural low-caffeine/high-theobromine tea resource.

## 1. Introduction

As an ancient and popular non-alcoholic beverage second only to water, tea is globally renowned for its desirable flavor profile and potential health benefits [1]. It possesses an abundance of bioactive compounds, predominantly polyphenols, theanine, alkaloids, and volatile components. Catechins constitute the primary polyphenols, whereas caffeine, theobromine, and theophylline represent the principal alkaloids; together, these metabolites impart unique sensory qualities and physiological activities to tea. Notably, the concentrations of these major bioactive constituents vary significantly among different tea germplasms. For instance, within representative Chinese tea plant germplasms, catechin levels can be lower than 60 mg/g in low-content varieties, whereas they exceed 200 mg/g in high-content varieties [2]. Caffeine is typically the most abundant alkaloid, with its average content ranging from 27 to 43 mg/g across various tea types [3]. In addition, volatile compounds play a crucial role in the formation of tea aroma.

Botanically, tea belongs to Sect. *Thea* (L.) Dyer within the genus *Camellia* L. of the family *Theaceae*. The taxonomic system proposed by H. T. Chang (Zhang Hongda) is among the most widely recognized and universally applied frameworks for the genus *Camellia* [4,5,6,7]. In this system, based on floral morphological characteristics, such as the presence or absence of ovary pubescence, the number of ovary locules, and the number of stigma lobes, Sect. *Thea* is further divided into four series: Ser. *Quinquelocularis*, Ser. *Pentastylae*, Ser. *Gymnogynae*, and Ser. *Sinenses*. Among these, tea plants within Ser. *Quinquelocularis* and Ser. *Pentastylae* are relatively primitive, mostly wild types; those of Ser. *Sinenses* are more evolutionarily advanced, mainly cultivated type; while Ser. *Gymnogynae* exhibits intermediate traits between them. Currently, *Camellia sinensis* (L.) O. Kuntze is the most widely cultivated and utilized tea species worldwide [8]. In addition, Sect. *Thea* contains numerous wild relatives that constitute an essential reservoir of germplasm resources. Although closely related to *C. sinensis*, some of these wild relatives have superior traits rarely found in cultivated species, such as rich genetic diversity and distinct metabolic profiles [9,10]. Consequently, they hold significant value for the genetic breeding and cultivar improvement of tea plants.

Tea plants originated in the southwestern Chinese provinces of Yunnan, Guizhou, and Sichuan. Situated at the junction of Yunnan and Guizhou, Guangxi Province falls within the subtropical monsoon climate zone, characterized by a warm climate and abundant rainfall. These favorable conditions provide an ideal environment for tea plant growth, establishing the region as a secondary center for tea domestication and cultivation that harbors rich and diverse tea plant germplasms [11]. Recently, a novel and scientifically unidentified wild tea plant resource was discovered in Shizhai Town, Cangwu County, Wuzhou City, Guangxi Province, China. Because its leaves resemble those of bamboo, local villagers have named this plant “Zhuyecha” (ZYC, *Camellia* sp.). However, the lack of in-depth botanical and phytochemical investigations into ZYC has limited its further development.

To comprehensively understand and effectively utilize ZYC, this study initially investigated its morphological characteristics to clarify its botanical traits. Subsequently, typical individual plants of ZYC were selected and compared with three locally cultivated tea varieties, namely Hongyan 12 (HY12), Fuyun 6 (FY6), and Yinghong 9 (YH9), through metabolomic analysis to explore the chemical composition of ZYC. Furthermore, the major biochemical constituents of ZYC were quantified to elucidate its phytochemical properties. Additionally, the volatile components were analyzed to identify the key aroma compounds and major aroma profiles of ZYC. The present study provides theoretical support for further research, development, and utilization of ZYC, and highlights its important potential value in production and application.

## 2. Materials and Methods

### 2.1. Plant Materials

This study utilized the wild tea germplasm “Zhuyecha” (ZYC, *Camellia* sp.), located in Shizhai Town, Cangwu County, Wuzhou City, Guangxi Province, China, as the research material. For phytochemical investigations, twelve typical individual plants of ZYC were selected and designated as DC1–DC12. Subsequently, a comparative analysis was conducted using three local tea cultivars as the control (CK) group, specifically *Camellia sinensis* ‘Hongyan 12’ (HY12), *Camellia sinensis* ‘Fuyun 6’ (FY6), and *Camellia sinensis* ‘Yinghong 9’ (YH9). All harvested fresh tea leaves were immediately frozen in liquid nitrogen and subjected to vacuum freeze-drying. The freeze-dried leaves were then ground into a fine powder and stored at −80 °C for subsequent analysis.

### 2.2. Chemicals

Milli-Q ultrapure water was used during the experiment. Chromatography-grade *N*,*N*-dimethylformamide and methanol were purchased from TEDIA Company (Fairfield, OH, USA). Chromatography-grade formic acid, acetonitrile, and acetic acid were obtained from Sinopharm Chemical Reagent Co., Ltd. (Shanghai, China). Epigallocatechin gallate (EGCG, ≥98%), epigallocatechin (EGC, ≥98%), epicatechin gallate (ECG, ≥98%), epicatechin (EC, ≥98%), catechin (C, ≥98%), and gallic acid (GA, ≥98%) were purchased from Shanghai Yuanye Bio-Technology Co., Ltd. (Shanghai, China). Caffeine (CAF, ≥98%) was acquired from Enzo Life Science (Farmingdale, NY, USA), while theobromine (TB, ≥98%) and theophylline (TP, ≥98%) were sourced from Shanghai Macklin Biochemical Co., Ltd. (Shanghai, China). Sodium chloride (NaCl), ethyl decanoate (≥99%), and the n-alkane standard mixture (C7–C40) were obtained from Sinopharm Chemical Reagent Co., Ltd., Shanghai Macklin Biochemical Co., Ltd., and Anpel Laboratory Technologies (Shanghai, China), respectively.

### 2.3. Morphological Characteristics Analysis

In accordance with the “Descriptors and data standard for tea (*Camellia* spp.)” [12], the “Technical code for evaluating crop germplasm tea plant (*Camellia sinensis*)” (NY/T 1312-2007), and the “Guidelines for the conduct of tests for distinctness, uniformity and stability—tea (*Camellia sinensis* (L.) O. Kuntze)” (NY/T 2422-2013), the botanical morphological features of ZYC were observed, recorded, and described, including plant, leaf, young shoot, flower, fruit, and seed.

### 2.4. Non-Volatile Metabolomics Analysis

Tea sample preparation: 0.1 g of tea powder was extracted with 20 mL of 70% (*v*/*v*) methanol. The mixture was incubated in a water bath at 70 °C for 30 min, with shaking every 10 min, and then centrifuged at 8000 r/min for 10 min. The resulting supernatant was filtered through a 0.22 μm membrane for non-volatile metabolomics analysis. Each sample was prepared in three independent biological replicates. Quality control (QC) samples were prepared by pooling equal aliquots from each sample.

UPLC-MS/MS conditions: Metabolomics analysis of tea samples was conducted using a UPLC-Q-Exactive-Orbitrap-MS/MS system (Thermo Fisher Scientific, Waltham, MA, USA). Chromatographic separation was achieved on an ACQUITY UPLC HSS T3 column (2.1 mm × 100 mm, 1.8 μm; Waters, Manchester, UK). The column temperature was maintained at 40 °C, the flow rate was 0.4 mL/min, and the injection volume was 3 μL. The mobile phase consisted of 0.1% formic acid in water (A) and 0.1% formic acid in acetonitrile (B). The elution program was as follows: 2% B (0–0.5 min), 15% B (0.5–8 min), 35% B (8–13 min), 70% B (13–15 min), 85% B (15–16 min), 2% B (16–16.5 min), and 2% B (16.5–20 min). MS detection was performed using electrospray ionization (ESI) in both positive and negative ion modes. The parameters were set as follows: the capillary voltage was 3500 V for positive mode and −3000 V for negative mode; the capillary temperature was maintained at 300 °C; the flow rate and temperature of drying gas were 10 L/min and 325 °C, respectively; and the mass scan range was 100–1200 *m*/*z*.

Data processing: The acquired raw data were processed using Compound Discoverer 3.2 (Thermo Fisher Scientific) for peak extraction and alignment. Metabolite identification was conducted based on standards, retention times, accurate masses, MS^2^ spectra, metabolomics databases (Metlin and HMDB), and previous work [13,14].

### 2.5. Absolute Quantification of Catechins, Alkaloids, and Gallic Acid

Sample preparation: 0.1 g of tea powder was extracted with 10 mL of 70% (*v*/*v*) methanol for 5 min, with shaking every 1 min. After centrifugation at 5000 r/min for 20 min, 2 mL of the supernatant was filtered through a 0.45 μm membrane for quantitative analysis by high-performance liquid chromatography (HPLC). Each sample was analyzed in three biological replicates.

HPLC conditions: Quantification was conducted on a Prominence LC-20A system (Shimadzu, Kyoto, Japan) equipped with a Welchrom-C18 column (4.6 mm × 250 mm, 5 μm; Welch, Shanghai, China) via the external standard method. The column temperature was maintained at 35 °C, the flow rate was 1 mL/min, and the UV detection wavelength was set at 278 nm. The injection volume was 20 μL. The mobile phase A consisted of ultrapure water, while mobile phase B comprised a mixture of *N*,*N*-dimethylformamide, methanol, and acetic acid (39.5:2:1.5, *v*/*v*/*v*). The following elution gradient was applied: 14–23% B (0–13 min), 23–36% B (13–28 min), 36% B (28–31 min), 36–14% B (31–34 min), and 14% B (34–43 min).

### 2.6. Volatile Component Analysis

Sample preparation and extraction: Volatile components were extracted by headspace solid-phase microextraction (HS-SPME). Firstly, 0.5 g of tea powder was accurately weighed into a 20 mL headspace vial, supplemented with 0.5 g of NaCl, 5 mL of boiled ultrapure water, and 10 μL of ethyl decanoate (used as an internal standard). Subsequently, the vial was equilibrated on a heating plate at 80 °C for 10 min. Following equilibration, extraction was performed using a 50/30 μm DVB/CAR/PDMS fiber (Supelco, Bellefonte, PA, USA) for 50 min, which was then desorbed for 5 min. The fiber was aged for 25 min before extraction, and each sample was processed in three biological replicates.

GC-MS conditions: Helium (99.999% purity) served as the carrier gas at a constant flow rate of 1.0 mL/min under splitless injection mode. The injection port temperature was set at 250 °C. The oven temperature program was established as follows: an initial temperature of 50 °C for 5 min, followed by a ramp to 80 °C at a rate of 2 °C/min (held for 1 min), an increase to 150 °C at 3 °C/min (held for 1 min), a subsequent elevation to 180 °C at 5 °C/min, and a final ramp to 230 °C at 15 °C/min (maintained for 5 min). Mass spectrometry was employed in electron impact ionization (EI) mode at 70 eV. The ion source temperature was 230 °C and the mass scan range was 35–400 *m*/*z*.

Identification and quantification of volatile compounds: The qualitative identification of all detected volatile compounds was accomplished by matching their mass spectra and calculated retention indices (RIs) against standard references within the NIST 22.0 library. The RIs of the volatiles were calculated based on the retention times of an n-alkane standard mixture (C7–C40) analyzed under identical GC-MS conditions. A compound was considered positively identified when the spectral similarity was high and the absolute difference between the calculated RIs and reference RIs was less than 20 [15]. Relative quantification was performed using ethyl decanoate as the internal standard.

Odor activity values (OAVs) analysis: The OAV was calculated by dividing the relative content of each volatile compound by its odor threshold in water to assess the contribution of the compound to the overall tea aroma. The odor thresholds used in this study were obtained from existing literature [16,17,18,19,20,21,22,23,24].

### 2.7. Data Analysis

For quantitative biochemical and metabolomic analyses, all tea samples were prepared in three biological replicates. The acquired data were processed using Excel 2019 (Microsoft, Redmond, WA, USA), and one-way analysis of variance (ANOVA) was performed with SPSS 25 (IBM, Armonk, NY, USA), followed by Duncan’s multiple range test for post hoc comparisons. A threshold of *p* < 0.05 was considered to indicate a statistically significant difference. Multivariate statistical analysis, including principal component analysis (PCA), hierarchical cluster analysis (HCA), and partial least squares-discriminant analysis (PLS-DA), was conducted using SIMCA 14.1 (Umetrics, Umeå, Sweden). Scaling normalization was applied for PCA and PLS-DA. Bar plots were constructed using Origin 2018 (OriginLab, Northampton, MA, USA). Heatmaps illustrating differential non-volatile metabolites and key aroma compounds were generated using MultiExperiment Viewer 4.9.0 (Oracle, Redwood, CA, USA), with the data normalized by the Z-score method.

## 3. Results

### 3.1. Morphological Characteristics of ZYC

**Figure 1:**
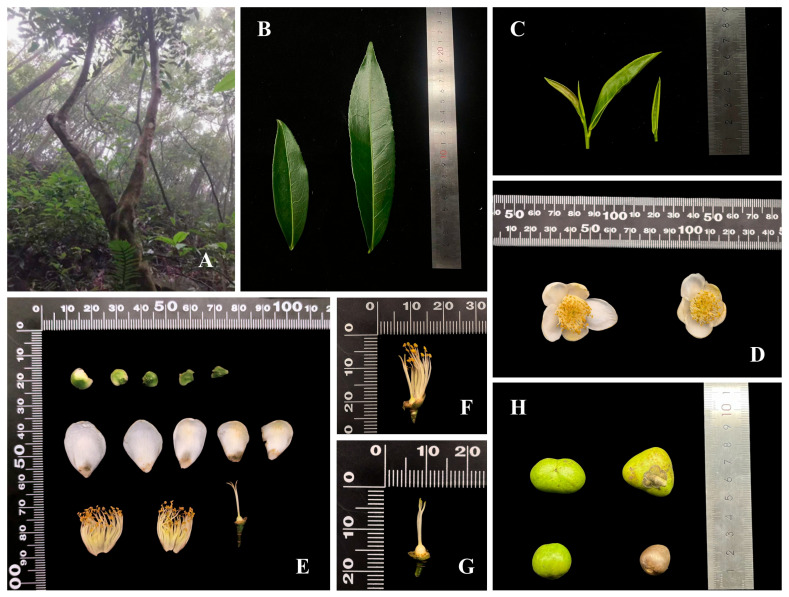
Morphological characteristics of Zhuyecha (ZYC). (**A**) A typical plant; (**B**) Leaves; (**C**) Young shoots; (**D**) Flowers; (**E**) A dissected flower; (**F**) Stamen and pistil of a flower; (**G**) Typical glabrous ovary and trifid style; (**H**) Fruits and seeds. Scale units of the rulers: cm for (**B**,**C**,**H**); mm for (**D**–**G**).

ZYC plants exhibit vigorous growth, with an arbor type, an upright habit, and a moderate branching density (Figure 1A). The leaves are green, mostly folded inward in cross-section, and the upper surface is flat; the leaf length is 10.1–19.0 cm, the leaf width is 2.5–4.5 cm, and the leaves are mainly large and medium-sized; the leaf apex is acuminate, the leaf base is cuneate, and the leaf shape is lanceolate (Figure 1B). The spring shoots have robust buds; the buds and leaves are sparsely pubescent or glabrous (Figure 1C). The peak blooming period of ZYC is in mid-October, and the corolla diameter is 2.5–3.2 cm (Figure 1D). Each flower has 5 sepals with pubescence on the outer side, and 5 petals measuring 0.6–1.7 cm in width, which are white on the inner surface (Figure 1E). The style is trifid, the stigma lobes are equal in height, and the ovary is smooth and glabrous (Figure 1F,G). The fruits are primarily spherical, reniform, or triangular in shape, 1.9–2.7 cm in diameter; the seeds are mostly spherical, 0.8–1.4 cm in diameter (Figure 1H).

### 3.2. Differential Analysis of Non-Volatile Metabolites Between ZYC Individual Plants and CK Cultivars

**Figure 2:**
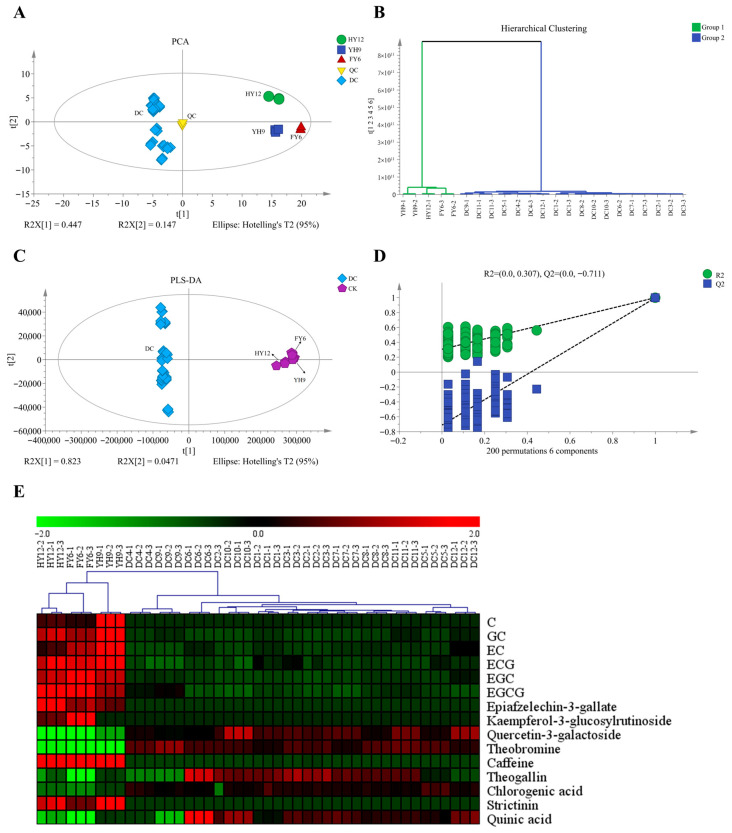
Non-volatile metabolite analysis of ZYC individual plants (DC1–DC12) and CK cultivars (HY12, FY6, and YH9). (**A**) PCA score plot; (**B**) Hierarchical cluster analysis (HCA) dendrogram calculated with ward’s method; (**C**) PLS-DA score plot; (**D**) cross-validation plot of PLS-DA model with 200 permutation tests (intercept: *R*^2^ = 0.307, *Q*^2^ = −0.711). (**E**) Heatmap of differential non-volatile metabolites. The color scale indicates Z-score normalized values of peak areas of metabolites, where green represents lower relative abundance and red represents higher relative abundance. ZYC, Zhuyecha (twelve individual plants: DC1–DC12); CK, control (three tea cultivars: HY12, ‘Hongyan 12’; FY6, ‘Fuyun 6’; YH9, ‘Yinghong 9’).

A non-targeted metabolomic analysis was performed on all fresh leaf samples using UPLC-MS/MS. A total of 159 non-volatile metabolites were identified, mainly encompassing flavanols, flavanol/flavone-O-glycosides, alkaloids, phenolic acids, organic acids, amino acids, lipids, and dimeric catechins, among other classes. The PCA results showed that QC samples clustered tightly at the center of the score plot (Figure 2A), suggesting good reproducibility of the metabolomic analysis. To further validate the instrumental stability, an empirical cumulative distribution function (ECDF) plot was generated. The results revealed that over 85% of the metabolites in the QC samples exhibited a coefficient of variation (CV) below 0.3 (Figure S1), confirming high data reliability. In the PCA score plot, the first two principal components (PC1 and PC2) explained 44.7% and 14.7% of the total variance, respectively. A clear separation was observed between the ZYC individuals (DC1–DC12) and the control cultivars (HY12, FY6, and YH9) primarily along the PC1 axis; the ZYC group was distributed in the second and third quadrants, whereas the control group was located in the first and fourth quadrants (Figure 2A). This indicates a significant shift in the overall metabolic profiles between the ZYC and CK samples. Hierarchical cluster analysis (HCA) was further performed using Euclidean distance as the similarity metric and Ward’s method as the clustering algorithm. The resulting dendrogram displayed a massive branching distance at the primary node, dividing the samples into two major clades: Group 1 (three control cultivars) and Group 2 (12 ZYC individuals) (Figure 2B). Subsequently, a supervised PLS-DA model was applied, which maximized the group separation and confirmed the marked metabolic differences between ZYC and CK (Figure 2C). A 200-permutation test yielded intercepts of 0.307 for *R*^2^ and −0.711 for *Q*^2^, demonstrating that the PLS-DA model was not overfitted (Figure 2D).

To elucidate the metabolic differences between ZYC and the three control cultivars, 15 differential non-volatile metabolites were screened in accordance with variable importance in projection (VIP) values exceeding 1 (Figure 2E). These comprised 7 flavanols, 3 phenolic acids, 2 alkaloids, 2 flavonol-O-glycosides, and 1 organic acid. Notably, two purine alkaloids, caffeine and theobromine, exhibited the highest VIP values, indicating that divergence in purine alkaloid metabolism acts as the principal biological factor discriminating the ZYC germplasm from cultivated varieties. Heatmap analysis further revealed that the tea samples were distinctly clustered into two groups, ZYC and CK (Figure 2E), which was consistent with the results of PCA, HCA, and PLS-DA (Figure 2A–C). Compared with CK cultivars (HY12, FY6, and YH9), ZYC contained higher contents of quercetin-3-galactoside, theobromine, theogallin, chlorogenic acid, and quinic acid. Conversely, the levels of catechins (C, GC, EC, ECG, EGC, and EGCG), epiafzelechin-3-gallate, kaempferol-3-glucosylrutinoside, caffeine, and strictinin were relatively lower in the ZYC group (Figure 2E).

### 3.3. Comparison of Major Chemical Components Between ZYC Individual Plants and CK Cultivars

**Figure 3:**
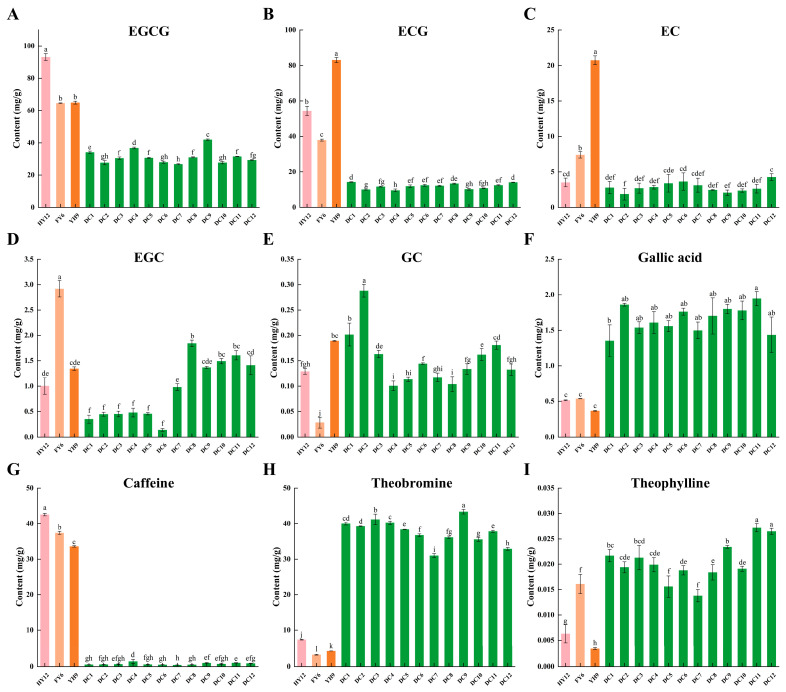
The contents of catechins, alkaloids, and gallic acid in ZYC individual plants (DC1–DC12) and CK cultivars (HY12, FY6, and YH9) determined by HPLC. (**A**) Epigallocatechin gallate (EGCG); (**B**) Epicatechin gallate (ECG); (**C**) Epicatechin (EC); (**D**) Epigallocatechin (EGC); (**E**) Gallocatechin (GC); (**F**) Gallic acid; (**G**) Caffeine; (**H**) Theobromine; (**I**) Theophylline. Data are presented as mean ± standard deviation (SD), *n* = 3. Different lowercase letters above the bars indicate significant differences at *p* < 0.05 (one-way ANOVA followed by Duncan’s multiple range test). ZYC, Zhuyecha (twelve individual plants: DC1–DC12); CK, control (three tea cultivars: HY12, ‘Hongyan 12’; FY6, ‘Fuyun 6’; YH9, ‘Yinghong 9’).

To further investigate the disparities in major chemical components between ZYC and the three cultivated varieties, HPLC was employed for absolute quantification of catechin monomers, three purine alkaloids, and gallic acid (Table S1). The total catechin content in ZYC was 40.42–55.82 mg/g, significantly lower than that in HY12 (152.12 ± 5.73 mg/g), FY6 (112.87 ± 0.95 mg/g), and YH9 (170.33 ± 1.90 mg/g) (Table S1). The concentrations of EGCG (26.81–41.99 mg/g) and ECG (9.62–14.30 mg/g) in ZYC were markedly reduced in contrast to those in the three control cultivars (exceeding 60 and 35 mg/g, respectively) (Figure 3A,B). The EC content in ZYC (1.88–4.27 mg/g) was generally lower than that in the CK cultivars and comparable to that in HY12 (Figure 3C). For EGC, the levels in ZYC individuals DC7–DC12 (0.98–1.84 mg/g) were relatively close to those of the control samples, while the concentrations in DC1–DC6 (0.14–0.48 mg/g) were notably lower (Figure 3D). In HY12, FY6, and ZYC, EGCG was the most abundant catechin; in contrast, ECG constituted the predominant catechin in YH9, in line with previous research [25]. The fresh leaves of ZYC were primarily composed of *epi*-form catechins, mainly EGCG and ECG, similar to the three cultivated varieties. It is noteworthy that the non-*epi*-form catechin GC reached the highest content in DC2 (0.29 ± 0.01 mg/g), and its overall level in ZYC was higher than that in the CK group (Figure 3E). Additionally, the gallic acid content in ZYC ranged from 1.35 to 1.95 mg/g, significantly surpassing that in the controls (2- to 5-fold) (Figure 3F).

Surprisingly, ZYC contained a remarkably high level of theobromine, reaching 31.01–43.37 mg/g, which was approximately 4- to 13-fold that of the controls (Figure 3H). Conversely, its caffeine content was extremely low, reaching only 1.40 mg/g at maximum (DC4), whereas all samples in the CK group exceeded 30 mg/g (Figure 3G). Moreover, the theophylline concentration was higher in ZYC than in the CK cultivars (Figure 3I). In terms of alkaloid composition, theobromine predominated in ZYC (96.58–98.60% of the total of the three), followed by caffeine (1.35–3.37%) and theophylline (0.04–0.08%). This unique profile is distinctly different from the caffeine-dominated composition pattern observed in the control varieties and other common tea cultivars [9].

### 3.4. Identification of Volatile Components

**Figure 4:**
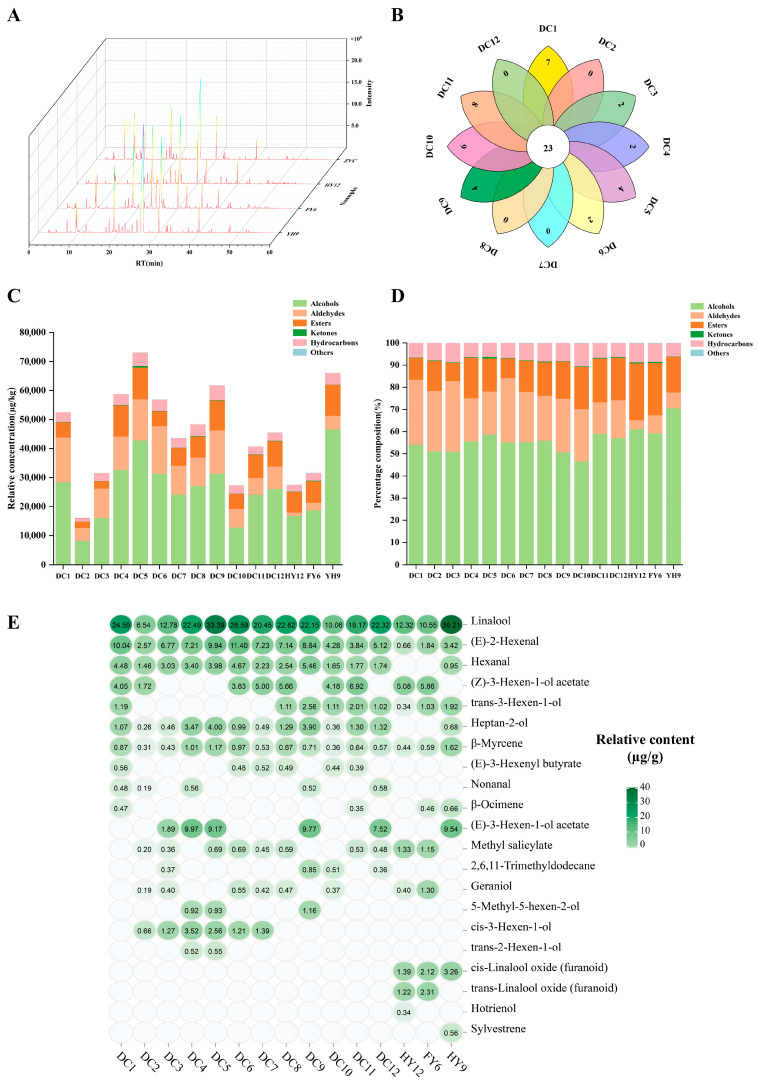
Identification of volatile components in ZYC individual plants (DC1–DC12) and CK cultivars (HY12, FY6, and YH9). (**A**) Representative total ion chromatograms; (**B**) Venn diagram of 12 ZYC individuals; Stacked bar plots of relative concentration (**C**) and percentage composition (**D**) for each volatile category; (**E**) Top 10 volatile compounds by relative content in different tea samples. The numerical values in the circles represent relative contents of corresponding compounds. The darker the green color, the higher the content. ZYC, Zhuyecha (twelve individual plants: DC1–DC12); CK, control (three tea cultivars: HY12, ‘Hongyan 12’; FY6, ‘Fuyun 6’; YH9, ‘Yinghong 9’).

The volatile profiles of 12 ZYC individuals and CK fresh tea leaves were characterized and compared via HS-SPME-GC-MS (Figure 4A). In total, 196 volatile compounds were identified across the 15 samples. The 12 ZYC individuals collectively contained 150 compounds, among which DC10 showed the highest number (85 compounds). The control cultivars HY12, FY6, and YH9 contained 88, 89, and 54 identified compounds, respectively. Across all samples, the volatile metabolites were classified into six categories: 49 alcohols, 23 aldehydes, 23 esters, 9 ketones, 83 hydrocarbons, and 9 others. In ZYC, the volatiles comprised 29 alcohols, 18 aldehydes, 22 esters, 8 ketones, 67 hydrocarbons, and 6 others. The Venn diagram (Figure 4B) indicated that 23 components were shared among all 12 ZYC individuals, including hexanal, (*E*)-2-hexenal, heptan-2-ol, 1-octen-3-ol, *β*-myrcene, *β*-ocimene, 6-methyl-5-hepten-2-one, *cis*-linalool oxide (furanoid), linalool, geraniol, methyl salicylate, decanal, tridecane, and *β*-cyclocitral.

The relative concentrations and proportional distributions of these volatile categories are presented in Figure 4C,D. Alcohols, which play an important role in tea aroma formation, accounted for the largest proportion (46.56–70.51%) in all fresh leaf samples. In addition, significant variation in total volatile content was observed across the samples (Figure 4C). Among the 15 tea samples, DC5 exhibited the highest concentration (73,013.53 ± 7581.41 μg/kg), followed by YH9 (66,034.59 ± 13,174.54 μg/kg). Except for DC2, which had a low content (16,050.14 ± 2692.00 μg/kg), other ZYC individuals reached volatile levels comparable to or higher than those of HY12 (27,525.07 ± 6296.65 μg/kg) and FY6 (31,618.72 ± 5361.66 μg/kg). These results suggested that ZYC fresh leaves contained a relatively higher abundance of volatile compounds. As shown in Figure 4D, ZYC had high proportions of alcohols, aldehydes, and esters, while the controls primarily consisted of alcohols and esters. This finding further indicated that ZYC may possess a distinct aroma profile compared with the control samples.

The relative contents of the top 10 volatile components in the ZYC and CK groups are shown in Figure 4E. These top 10 compounds accounted for over 85% of the total volatile content. Linalool, as a common aromatic constituent in tea, was the most abundant across all samples, with its content basically exceeding twice that of the second-ranking compound. Remarkably, the top 10 volatiles in all 12 ZYC individuals consistently included linalool, (*E*)-2-hexenal, hexanal, heptan-2-ol, and *β*-myrcene, all of which were also prominent in YH9.

To better characterize and differentiate the aroma constituents of the various tea samples, multivariate analysis was conducted on the volatile compounds identified by GC-MS, including PCA and PLS-DA (Figure S2). The analysis revealed a clear separation between the CK and ZYC samples. Moreover, the 12 individuals of ZYC were not completely clustered but rather scattered across four quadrants, reflecting a certain degree of inter-individual variation in the aroma metabolic profile within the population. The PLS-DA model was confirmed to be reliable after cross-validation with 200 permutation tests (intercepts of *R*^2^ and *Q*^2^ were 0.357 and −0.818, respectively). Subsequently, 46 differential volatile compounds were screened based on the criteria of VIP > 1. Among these, 12 compounds had VIP values exceeding 2, listed in descending order as follows: linalool, (*E*)-2-hexenal, (*Z*)-3-hexen-1-ol acetate, hexanal, *trans*-3-hexen-1-ol, (*E*)-3-hexen-1-ol acetate, *cis*-3-hexen-1-ol, *cis*-linalool oxide (furanoid), heptan-2-ol, *trans*-linalool oxide (furanoid), (*Z*)-2-hexen-1-ol acetate, and nonanal. They are important discriminatory substances that distinguish the metabolic profiles among the different groups.

### 3.5. Odor Activity Values (OAVs) Analysis

The odor activity value (OAV) can be used to evaluate the contribution of individual volatile components to the overall aroma. It is generally accepted that compounds with OAV > 1 contribute to the aroma, while those with OAV > 10 are considered to have a significant impact on the aroma profile [26]. Based on the concentrations of the identified volatiles and their reported thresholds in the literature, the OAVs were calculated and analyzed. As shown in Table 1, 39 volatile compounds had OAVs greater than 1. In the ZYC samples, 14 compounds fell within the range of 1 < OAV ≤ 10, including *cis*-*β*-ocimene, (*E*,*E*)-2,4-hexadienal, *cis*-linalool oxide (furanoid), *o*-cymene, tridecane, 2-heptanone, naphthalene, *trans*-2-Hexen-1-ol, 2,2,4-trimethyl-1,3-pentanediol diisobutyrate, *cis*-3-hexen-1-ol, *trans*-linalool oxide (furanoid), undecanal, 6-methyl-5-hepten-2-one, and D-limonene; 11 compounds within 10 < OAV ≤ 100, encompassing *β*-myrcene, geraniol, heptan-2-ol, dodecanal, *trans*-3-hexen-1-ol, 3-methylbutanal, methyl salicylate, *p*-cymene, *β*-ocimene, *β*-cyclocitral, and (*E*)-3-hexen-1-ol acetate; 7 compounds within 100 < OAV ≤ 1000, comprising (*E*)-2-hexenal, nonanal, 1-octen-3-ol, (*Z*)-3-hexen-1-ol acetate, 2,2,6-trimethylcyclohexanone, *trans*-2-nonenal, and decanal. Furthermore, 3 compounds had OAVs exceeding 1000: linalool, hexanal, and *trans*-*β*-ionone, which contributed the most to the overall aroma of fresh leaves. Among all ZYC individuals, linalool, hexanal, (*E*)-2-hexenal, *β*-myrcene, and heptan-2-ol consistently ranked in the top 10 by content, all with OAVs surpassing 10. Therefore, these specific constituents play an essential role in shaping the characteristic aroma profile of ZYC fresh leaves.

### 3.6. Key Aroma Compounds in ZYC

**Figure 5:**
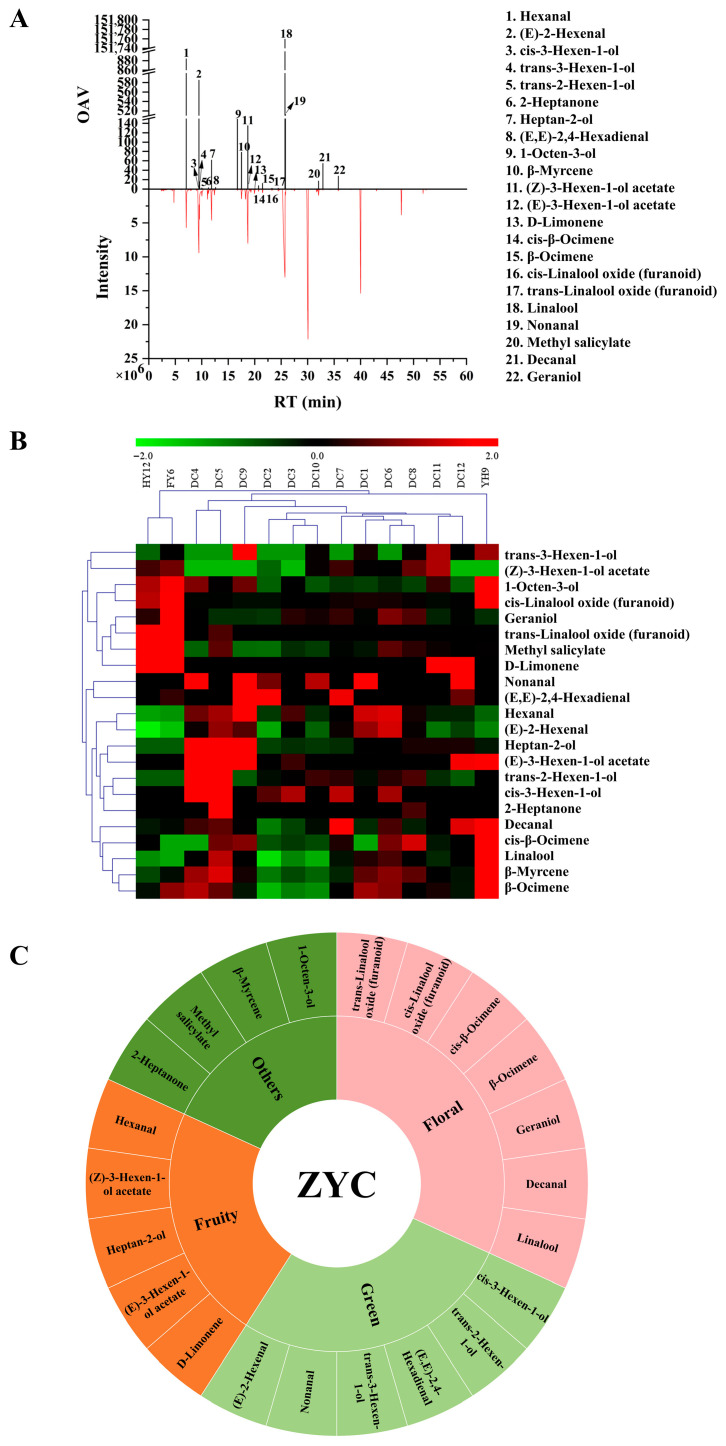
Key aroma compounds identified in ZYC. (**A**) Total ion intensities of the volatiles and their corresponding OAVs; (**B**) Heatmap of key aroma compounds across different tea samples (the color scale indicates Z-score normalized values of relative contents of volatile compounds, where green represents lower relative abundance and red represents higher relative abundance); (**C**) Aroma wheel of ZYC. ZYC, Zhuyecha (twelve individual plants: DC1–DC12); CK, control (three tea cultivars: HY12, ‘Hongyan 12’; FY6, ‘Fuyun 6’; YH9, ‘Yinghong 9’).

Based on the integrated criteria of OAV > 1, VIP > 1, and *p* < 0.05, a total of 23 volatile compounds were further screened out. Among them, 22 were detected in ZYC and identified as the key aroma compounds in ZYC fresh leaves, namely hexanal, (*E*)-2-hexenal, *cis*-3-hexen-1-ol, *trans*-3-hexen-1-ol, *trans*-2-hexen-1-ol, 2-heptanone, heptan-2-ol, (*E*,*E*)-2,4-hexadienal, 1-octen-3-ol, *β*-myrcene, (*Z*)-3-hexen-1-ol acetate, (*E*)-3-hexen-1-ol acetate, D-limonene, *cis*-*β*-ocimene, *β*-ocimene, *cis*-linalool oxide (furanoid), *trans*-linalool oxide (furanoid), linalool, nonanal, methyl salicylate, decanal, and geraniol (Figure 5A). The distribution of these key compounds across the different tea leaf samples was visualized using a clustered heatmap (Figure 5B). The resulting dendrogram revealed three distinct groups: one cluster contained HY12 and FY6 cultivars, another cluster comprised ZYC individuals, while YH9 clustered separately. In addition, statistically significant differences in the levels of specific compounds were observed among these groups. As illustrated in Figure 5B, the concentrations of geraniol, *trans*-linalool oxide (furanoid), and methyl salicylate were significantly higher in HY12 and FY6 than in the other groups; in comparison to the control cultivars, the ZYC group exhibited markedly increased levels of nonanal, (*E*,*E*)-2,4-hexadienal, hexanal, (*E*)-2-hexenal, heptan-2-ol, *trans*-2-hexen-1-ol, and *cis*-3-hexen-1-ol; whereas YH9 showed a prominent abundance of linalool, *β*-myrcene, *β*-ocimene, *cis*-*β*-ocimene, *cis*-linalool oxide (furanoid), and 1-octen-3-ol.

To further clarify the aroma profile, an aroma wheel of ZYC fresh leaves was constructed using the 22 key aroma components identified above (Figure 5C). According to their odor descriptions (Table 1), these compounds were categorized into four aroma attribute types: floral (7), fruity (5), green (6), and others (4). The key compounds contributing to the floral attribute mainly included linalool, geraniol, decanal, and *β*-ocimene; among these, linalool exhibited the highest OAV (>1000) of the 22 compounds, serving as the dominant contributor to the floral note of ZYC. Representative compounds associated with the fruity attribute included hexanal, (*Z*)-3-hexen-1-ol acetate, and heptan-2-ol. The green attribute was primarily represented by (*E*)-2-hexenal, nonanal, and *trans*-3-hexen-1-ol. The “others” category comprised compounds such as 1-octen-3-ol and *β*-myrcene.

## 4. Discussion

The present study provides the first detailed characterization of ZYC, a wild tea plant resource discovered in Shizhai Town, Cangwu County, Wuzhou City, Guangxi Province. Morphological analysis showed that ZYC is an arbor-type, upright plant bearing lanceolate leaves. The flower possesses five sepals, five petals, a trifid style, and a glabrous ovary. Such characteristics distinguish ZYC from conventionally cultivated tea plants, which are mostly small arbor or shrub types with an open or semi-open growth habit and usually have pubescent ovaries [9,27,28]. According to the Zhang Hongda taxonomic system, tea plants within Ser. *Gymnogynae* share specific morphological traits, such as glabrous ovaries and trifid styles, which serve as diagnostic features separating this series from other series within Sect. *Thea* [4]. Correspondingly, ZYC conforms to these typical characteristics. By searching relevant literature and materials for comparison, we found that ZYC shares morphological similarities with Rongjiangcha (*Camellia yungkiangensis*, RJC), Tulecha (*Camellia costata*, TLC), Tufangcha (*Camellia gymnogyna*, TFC), and the recently reported Yuanbaoshancha (*Camellia yungkiangensis* var. *yuanbaoshanica*, YBSC) [4,7,29,30]. Notably, RJC, TLC, and TFC are all classified under Ser. *Gymnogynae*, while YBSC is regarded as a variety of RJC. These wild tea relatives all have distribution records in Guangxi.

To fully develop and utilize this special tea resource, we further employed UPLC-MS/MS and HPLC to detect and analyze the chemical constituents. In this work, as a wild tea germplasm, ZYC exhibited significant differences from cultivated controls in alkaloid composition and catechin content, demonstrating a unique phytochemical signature. Compared with common tea cultivars, ZYC leaves had higher theobromine, extremely lower caffeine, higher theophylline, higher gallic acid, and lower total catechin contents. Intriguingly, the chemical profile of low caffeine and high theobromine observed in ZYC was also found in RJC, TLC, TFC and YBSC [29,30,31,32]. This finding may further support the taxonomic affinity of ZYC with Ser. *Gymnogynae*. It is worth noting that the catechin accumulation patterns differ among these wild tea plants. The content of catechin components in ZYC was as follows: EGCG > ECG > EC > EGC > GC, with *epi*-form/galloylated catechins predominating, a pattern similar to that of common cultivated varieties. In contrast, RJC and YBSC primarily accumulate the non-*epi*-form/non-galloylated catechin C, while TLC and TFC are dominated by the *epi*-form/galloylated catechin EGCG [25,29,30,31,33]. This is related to differences in the activities or expression levels of key enzymes in the catechin biosynthesis pathway. The metabolic flux towards *epi*-form versus non-*epi*-form catechins depends largely on the catalytic balance between anthocyanidin reductase (ANR) and leucoanthocyanidin reductase (LAR), with the upstream enzyme anthocyanidin synthase (ANS) supplying the necessary substrate for ANR [25,34]. Furthermore, the substantial accumulation of non-galloylated catechins in certain wild species may imply downregulation or functional variation in the key enzymes responsible for galloylation, such as serine carboxypeptidase-like acyltransferases (SCPL-ATs) [35].

Combined with the available morphological and phytochemical evidence, we preliminarily speculate that ZYC may belong to Ser. *Gymnogynae*. However, tea plants exhibit high genetic variability and are prone to natural hybridization [36]. Although the current morphological and chemical characteristics provide critical clues, these alone are often insufficient to resolve complex taxonomic ambiguities. Given the lack of molecular phylogenetic analysis, a definitive genetic basis has yet to be established. Therefore, the classification proposed herein serves as a preliminary inference. Future investigations employing advanced molecular tools, such as DNA barcoding (e.g., *ITS*, *matK*, or *rbcL*) and chloroplast genome sequencing, are highly warranted [9,37,38]. The inferred affinity with Ser. *Gymnogynae* in this study may provide a direction for subsequent phylogenetic reconstruction. Future molecular verification could compare ZYC with representative species of Ser. *Gymnogynae* and a broader range of closely related species within Sect. *Thea*, using typical cultivated varieties (*C. sinensis*) as genetic references. Ultimately, a comprehensive approach integrating morphological, chemical, and molecular biological evidence is required to determine the formal taxonomic status of this wild tea resource.

Caffeine is the primary alkaloid in most tea varieties, well known for its refreshing and fatigue-alleviating effects [8,39]. While moderate intake is generally beneficial, excessive consumption may cause insomnia, anxiety, arrhythmia, and other health risks [40,41]. Growing public health awareness has driven increasing market demand for low-caffeine or caffeine-free tea products, making the breeding of natural low-caffeine tea plants an important research objective [40]. Although low-caffeine tea resources remain scarce, continuous botanical exploration has uncovered several valuable breeding materials. In addition to RJC, TLC, TFC, and YBSC, the most representative resource is cocoa tea (*Camellia ptilophylla*). Originating from Guangdong Province, this species is rich in theobromine and GCG, nearly devoid of caffeine [39,42]. It has been scientifically proven to have various bioactivities, such as anti-inflammatory [43], anticancer [44], and anti-adipogenic properties [45]. Hongyacha, distributed in the mountainous regions of Fujian Province, accumulates theobromine and *trans*-catechins, and is considered beneficial for disease prevention or adjunctive therapy [46]. Similarly, the novel wild tea germplasm ZYC in this study exhibits a unique metabolic phenotype characterized by low caffeine and high theobromine levels. This is likely attributed to genetic variations in the purine alkaloid biosynthesis pathway, such as mutations or downregulated expression of the caffeine synthase gene (e.g., *TCS1*), which block the final methylation step converting theobromine to caffeine [46]. Therefore, in-depth research on its underlying molecular mechanisms will provide a crucial theoretical basis for improving existing tea varieties and breeding new low-caffeine cultivars.

The theobromine content in ZYC was significantly higher than that of common cultivated varieties (31.01–43.37 mg/g), whereas the caffeine content was extremely low (0.44–1.40 mg/g). As a structural analog of caffeine, theobromine exerts milder physiological activities and has a higher physiological effect threshold [47]. Previous pharmacological studies have shown that theobromine confers multiple positive effects, for instance, promoting fat metabolism [48], improving lipid profiles [49], inducing vasodilation [50], inhibiting inflammatory factors [51], and enhancing memory [52,53]. Notably, a clinical trial indicates that a single oral dose of 250 mg of theobromine is well-tolerated in humans, while high doses (500–1000 mg) can trigger adverse reactions, including negative mood changes and increased heart rate [54]. If tea made from ZYC is consumed via traditional brewing methods (assuming 3–5 g of dry tea per serving), the actual theobromine intake is expected to remain within the safe threshold. However, high-concentration and excessive consumption should be avoided.

Beyond theobromine, ZYC was found to be rich in other important bioactive compounds, such as gallic acid, chlorogenic acid, and quercetin-3-galactoside. Gallic acid is a significant natural polyphenol with well-documented antioxidant, anti-inflammatory, antibacterial, anticancer, and neuroprotective effects [55,56,57,58]. Chlorogenic acid, a phenolic acid synthesized through the plant phenylpropanoid pathway, exhibits antibacterial, anti-inflammatory, antioxidant, hypoglycemic, and cardiovascular protective activities [59,60]. Quercetin-3-galactoside, a flavonol glycoside, has also been reported to have various health benefits [61]. The synergistic interaction of these key functional compounds provides a material basis for the health value of ZYC. Meanwhile, these abundant biochemical components also determine the quality characteristics of tea. In ZYC fresh leaves, the levels of caffeine and catechins, which are responsible for strong bitterness and astringency, were significantly reduced, whereas the specifically accumulated theobromine conferred a relatively milder bitterness. Phenolic acids like gallic acid may impart a slight sourness and astringency to the tea infusion [62]. Given that the present study focused on the chemical profiling of fresh leaves, the comprehensive sensory quality of processed ZYC tea remains to be evaluated. Hence, future efforts could be directed toward processing optimization and finished tea research, with exploratory development of healthy functional low-caffeine tea beverages or distinctive flavor products. Overall, ZYC shows favorable market potential, particularly for caffeine-sensitive consumer groups, and also serves as a suitable raw material for the production of high-value natural products.

Driven by its unique physicochemical characteristics, each tea plant variety is typically predisposed to processing into specific tea types to achieve optimal sensory quality and commercial value [63]. The abundance and proportion of chemical components in fresh leaves directly influence their processing suitability [13]. Studies have shown that black or white teas manufactured from certain natural low-caffeine and high-theobromine tea resources exhibit excellent quality and remarkable bioactivities. For instance, black and white teas processed from RJC fresh leaves possess strong floral and fruity aromas, yielding higher sensory scores alongside greater antioxidant and hypoglycemic activities than traditional cultivars [31]. YBSC black tea has an extremely low caffeine content (0.20%) and a high theobromine content (3.98%), displaying a pleasant peach aroma and a smooth, mellow taste [30]. The antioxidant and anti-inflammatory effects of black cocoa tea are superior to those of black tea processed from *C. sinensis* [42,64]. Additionally, existing evidence indicates that theobromine and chlorogenic acid are relatively abundant in cultivars suitable for black tea production [13]. In this work, ZYC fresh leaves were characterized by low caffeine, high theobromine, high chlorogenic acid, and unique chemical constituents. This may endow ZYC with potential for quality formation and bioactivity during black tea processing.

Meanwhile, ZYC fresh leaves contained a large amount of volatile compounds (total content reaching up to 73,013.53 ± 7581.41 μg/kg). These mainly comprised alcohols, aldehydes, and esters, which formed a floral, fruity, and green aroma profile. Earlier studies have indicated that alcohols primarily contribute to floral aromas, aldehydes impart citrus and green notes, while esters provide sweet and coconut-like scents [65]. Notably, numerous key aroma compounds identified in ZYC, including geraniol, methyl salicylate, 1-octen-3-ol, linalool, hexanal, limonene, (*Z*)-3-hexen-1-ol acetate, *trans*-linalool oxide (furanoid), nonanal, and *β*-myrcene, have also been recognized as crucial odorants in four world-famous black teas [66]. Moreover, the terpenoids linalool, geraniol, and *β*-ionone carry distinct floral and sweet notes, which markedly contribute to the fragrance of black tea and oolong tea, including *Keemun* black tea [67], Congou black tea [68], *Camellia nanchuanica* black tea [69], and Rougui Wuyi rock tea [70]. (*R*)-linalool has been reported as a core flavor component and an important quality marker of YH9 black tea [71]. Taken together, we speculate that ZYC may have the potential for black tea production. Nevertheless, it must be acknowledged that the fermentation process significantly alters the chemical composition and aroma profile of fresh tea leaves. Therefore, subsequent systematic processing trials, combined with sensory evaluations and biochemical analyses, are still required to explore its optimal processing suitability.

## 5. Conclusions

This study characterized the novel wild tea resource ZYC from Guangxi Province through morphological, LC-MS/GC-MS metabolomic, and quantitative chemical analyses. Morphologically, ZYC is an arbor-type plant with an upright growth habit and lanceolate leaves. The flower has five petals and five sepals, with the glabrous ovary and trifid style serving as critical taxonomic features. Given the complexity of tea plant genetics and classification, its exact taxonomic status requires a comprehensive identification incorporating molecular biology techniques. Chemically, ZYC differed significantly from the control cultivated tea varieties (HY12, FY6, and YH9). ZYC fresh leaves contained higher levels of theobromine, theophylline, and gallic acid, but extremely lower caffeine content and lower total catechin content compared with the control cultivars. Theobromine dominated the alkaloid profile, while EGCG and ECG were the predominant catechins. In addition, important bioactive substances such as chlorogenic acid and quercetin-3-galactoside were identified in ZYC. Based on OAV > 1, VIP > 1, and *p* < 0.05, 22 key aroma compounds were screened from ZYC fresh leaves. Representative compounds included linalool, (*E*)-2-hexenal, hexanal, heptan-2-ol, *β*-myrcene, (*Z*)-3-hexen-1-ol acetate, nonanal, decanal, geraniol, and 1-octen-3-ol, contributing to its characteristic floral, fruity and green aroma notes. These results confirm that ZYC is a unique and promising tea germplasm characterized by naturally low caffeine, high theobromine, and abundant functional quality components. In summary, this study lays a fundamental foundation for future research on the exploration and utilization of ZYC germplasm.

## Figures and Tables

**Table 1:** Odor descriptions, thresholds and OAVs of volatile compounds in ZYC (DC1–DC12) and CK (HY12, FY6, and YH9) tea samples.

NO	Compounds	Category	RI ^1^	Odor Description ^2^	Threshold (μg/L)	OAV														
						DC1	DC2	DC3	DC4	DC5	DC6	DC7	DC8	DC9	DC10	DC 11	DC 12	HY 12	FY6	YH9
1	3-Methylbutanal	Aldehydes	656	Chocolate, Peach, Fatty	1.1	ND	ND	14.30	ND	ND	26.31	ND	ND	ND	ND	ND	ND	ND	ND	ND
2	Hexanal	Aldehydes	793	Apple, Fat, Fresh, Green, Oil	4.5	995.65	325.20	674.20	756.48	883.94	1037.54	495.22	563.48	1213.38	366.51	394.37	386.78	42.61	76.14	211.89
3	(*E*)-2-Hexenal	Aldehydes	844	Green, Banana, Fatty, Cheesy	17	590.73	151.29	398.18	423.98	584.86	670.44	425.38	419.72	508.50	251.65	225.65	301.00	38.94	108.15	201.00
4	*cis*-3-Hexen-1-ol	Alcohols	848	Grass, Green Fruit, Green Leaf, Herb, Unripe Banana	1900	ND	0.35	0.67	1.85	1.35	0.64	0.73	ND	ND	ND	ND	ND	ND	ND	ND
5	*trans*-3-Hexen-1-ol	Alcohols	849	Grass, Green Fruit, Green Leaf, Herb, Unripe Banana	70	16.97	ND	ND	ND	ND	ND	ND	15.92	36.59	15.80	28.74	14.51	4.83	14.69	27.46
6	*trans*-2-Hexen-1-ol	Alcohols	857	Fresh, Green Leaf, Fruity, Unripe Banana	231.9	0.42	0.41	0.60	2.24	2.39	0.76	0.78	0.93	ND	0.86	0.26	ND	ND	ND	0.53
7	2-Heptanone	Ketones	883	Blue Cheese, Fruit, Green, Nut, Spice	140	ND	ND	ND	ND	3.00	ND	ND	0.40	ND	ND	ND	ND	ND	ND	ND
8	Heptan-2-ol	Alcohols	894	Citrus, Earth, Fried, Mushroom, Oil	65.235	16.44	4.04	7.11	53.13	61.34	15.18	7.44	19.72	59.79	5.53	19.91	20.16	ND	ND	10.49
9	Heptanal	Aldehydes	897	Citrus, Fat, Green, Nut	2.8	ND	ND	ND	ND	ND	ND	ND	ND	ND	ND	ND	ND	13.12	22.50	ND
10	(*E*,*E*)-2,4-Hexadienal	Aldehydes	904	Green, Sweet, Fruity, Waxy, Fatty	10	ND	5.92	ND	ND	ND	ND	2.92	ND	3.61	ND	ND	1.27	ND	0.67	ND
11	(*E*)-2-Heptenal	Aldehydes	968	Almond, Fat, Fruit	1.1	ND	ND	ND	ND	ND	ND	ND	ND	ND	ND	ND	ND	ND	7.19	ND
12	1-Octen-3-ol	Alcohols	972	Cucumber, Earth, Fat, Floral, Mushroom	1	63.62	28.30	137.71	262.94	148.88	90.42	74.92	61.59	246.69	38.66	194.71	30.42	306.95	384.11	510.21
13	6-Methyl-5-hepten-2-one	Ketones	980	Citrus, Mushroom, Pepper, Rubber, Strawberry	68	1.05	0.34	0.54	1.19	1.21	1.09	0.66	0.82	1.12	0.44	1.15	1.55	0.53	0.88	0.72
14	*β*-Myrcene	Hydrocarbons	984	Balsamic, Fruit, Geranium, Herb, Must	15	58.23	20.34	29.00	67.52	78.02	64.66	35.22	58.14	47.48	24.15	42.71	38.33	29.25	39.21	107.97
15	(*Z*)-3-Hexen-1-ol acetate	Esters	1002	Banana, Candy, Floral, Green	31	130.78	55.52	ND	ND	ND	123.56	161.14	182.56	ND	134.82	223.26	ND	163.80	188.89	ND
16	(*E*)-3-Hexen-1-ol acetate	Esters	1005	Fruit	870	ND	ND	2.17	11.46	10.54	ND	ND	ND	11.23	ND	ND	8.65	ND	ND	10.97
17	*p*-Cymene	Hydrocarbons	1015	Citrus, Fresh, Solvent	5.01	15.95	3.86	6.30	9.47	11.14	12.53	ND	8.27	ND	3.63	6.09	ND	5.34	ND	11.83
18	*o*-Cymene	Hydrocarbons	1017	Aromatic	11.4	ND	ND	ND	ND	ND	ND	4.21	ND	ND	ND	ND	2.25	ND	ND	ND
19	D-Limonene	Hydrocarbons	1021	Citrus, Mint, Lemon, Orange-like, Green	200	ND	ND	ND	ND	ND	ND	ND	ND	ND	ND	1.13	1.02	1.16	0.89	ND
20	2,2,6-Trimethylcyclohexanone	Ketones	1026	Floral	0.1	145.35	ND	ND	186.97	128.17	163.58	161.13	88.15	216.89	129.44	186.21	101.21	ND	ND	ND
21	*cis*-*β*-Ocimene	Hydrocarbons	1029	Floral	34	ND	2.34	2.91	ND	7.26	7.45	5.45	9.69	7.75	2.51	3.81	4.59	4.58	ND	11.37
22	*β*-Ocimene	Hydrocarbons	1030	Floral	34	13.92	2.92	4.89	14.59	12.27	13.18	9.21	9.24	8.30	4.35	10.22	8.37	8.49	13.44	19.45
23	*γ*-Terpinene	Hydrocarbons	1050	Bitter, Citrus	55	ND	ND	ND	ND	ND	ND	ND	ND	ND	ND	ND	ND	ND	ND	1.76
24	*cis*-Linalool oxide (furanoid)	Alcohols	1064	Floral	100	4.50	1.15	2.47	1.62	1.74	4.40	3.99	3.47	1.11	2.22	1.27	2.09	13.91	21.15	32.59
25	*trans*-Linalool oxide (furanoid)	Alcohols	1082	Floral	190	ND	ND	ND	ND	1.85	ND	ND	ND	ND	ND	ND	ND	6.44	12.13	ND
26	Linalool	Alcohols	1099	Floral, Lavender, Lemon, Rose	0.22	111,784.98	29,741.11	58,102.16	102,224.44	151,759.17	120,854.00	92,967.18	102,838.62	100,670.89	45,714.19	87,141.12	101,449.29	55,988.81	47,934.13	178,206.55
27	Nonanal	Aldehydes	1099	Green, Grassy, Cucumber, Melon	1.1	435.52	176.02	ND	512.40	ND	ND	ND	ND	474.16	271.56	ND	523.32	ND	ND	ND
28	Hotrienol	Alcohols	1101	Floral, Fresh, Fruity	110	ND	ND	ND	ND	ND	ND	ND	ND	ND	ND	ND	ND	3.07	ND	ND
29	*trans*-2-Nonenal	Aldehydes	1169	Paper	0.19	ND	ND	ND	ND	ND	ND	77.59	ND	ND	ND	ND	109.16	ND	ND	ND
30	Naphthalene	Hydrocarbons	1174	Pungent, Dry, Tarry	6	ND	0.54	1.13	ND	ND	1.64	1.21	0.91	ND	0.84	2.83	0.78	ND	ND	ND
31	Methyl salicylate	Esters	1186	Almond, Caramel, Peppermint, Sharp	40	10.37	5.05	8.96	5.84	17.21	17.19	11.23	14.80	5.16	8.03	13.21	12.03	33.13	28.77	12.65
32	Decanal	Aldehydes	1198	Floral, Fried, Orange Peel, Penetrating, Tallow	3	27.02	10.32	22.13	49.73	54.34	51.24	108.06	29.71	39.37	32.80	37.10	78.96	30.91	33.70	94.83
33	*β*-Cyclocitral	Aldehydes	1212	Herbal, Rose, Sweet, Fruity	3	7.02	3.89	4.40	11.73	9.84	10.21	10.78	7.84	7.91	9.42	7.57	7.38	ND	8.84	ND
34	Geraniol	Alcohols	1249	Floral, Sweet, Rose, Fresh, Fruity	7.5	38.64	25.93	53.26	41.91	27.43	73.18	56.22	62.68	27.46	49.48	30.62	39.20	53.63	173.39	27.62
35	Tridecane	Hydrocarbons	1294	Alkane	42	0.82	0.42	1.43	1.42	1.78	0.92	1.53	1.83	3.40	1.66	1.10	1.43	0.91	0.96	ND
36	Undecanal	Aldehydes	1300	Waxy, Floral, Citrus, Green, Fatty, Fresh	12.5	ND	ND	ND	0.73	0.89	0.74	1.52	ND	ND	0.61	0.88	1.67	0.60	0.37	1.57
37	Dodecanal	Aldehydes	1403	Citrus, Fat, Lily	0.29	ND	ND	ND	ND	ND	ND	ND	ND	ND	16.19	12.68	38.13	ND	ND	ND
38	*trans*-*β*-Ionone	Ketones	1478	Floral, Violet	0.007	ND	538.19	1547.45	2250.74	ND	1312.62	1290.62	1208.81	ND	1760.39	816.77	1337.88	ND	ND	1047.64
39	2,2,4-Trimethyl-1,3-pentanediol diisobutyrate	Esters	1591	-	14	1.14	0.64	ND	1.53	1.13	1.00	ND	2.34	ND	ND	0.91	0.90	1.97	1.72	1.19

ND, the compound was not detected; ^1^ RI, retention index; ^2^ odor descriptions were found in https://www.femaflavor.org/flavor-library (accessed on 14 July 2025) or https://www.thegoodscentscompany.com (accessed on 14 July 2025). ZYC, Zhuyecha (twelve individual plants: DC1–DC12); CK, control (three tea cultivars: HY12, ‘Hongyan 12’; FY6, ‘Fuyun 6’; YH9, ‘Yinghong 9’).

## Data Availability

Data are included in this article or Supplementary Materials.

## Supplementary Materials

The following supporting information can be downloaded at: https://www.mdpi.com/article/10.3390/plants15111642/s1. Figure S1: Empirical cumulative distribution function (ECDF) plot of the coefficient of variation (CV) for quality control (QC) samples in non-volatile metabolite analysis. Figure S2: Multivariate statistical analysis of volatile metabolites in ZYC individual plants (DC1–DC12) and CK cultivars (HY12, FY6, and YH9). From top to bottom: PCA score plot, PLS-DA score plot, cross-validation plot of PLS-DA model with 200 permutation tests (intercept: *R*^2^ = 0.357, *Q*^2^ = −0.818). ZYC, Zhuyecha (twelve individual plants: DC1–DC12); CK, control (three tea cultivars: HY12, ‘Hongyan 12’; FY6, ‘Fuyun 6’; YH9, ‘Yinghong 9’). Table S1: Quantitative analysis of alkaloids, gallic acid and catechins in ZYC individual plants (DC1–DC12) and CK cultivars (HY12, FY6, and YH9) determined by HPLC (mean ± SD, mg/g).
