# Supplementary material for: Morphological and Chemical Characterization of a Novel Wild Tea Plant Resource with Naturally Low Caffeine and High Theobromine from Guangxi Province, China
Source: Plants (Basel). 2026 May 27;15(11):1642. doi: 10.3390/plants15111642 (PMC13259505; doi:10.3390/plants15111642)
Supplement: Supplementary file 1 [file plants-15-01642-s001.zip › Supplementary Material-Proofread/Supplementary Material-1.pdf]

Morphological and Chemical Characterization of a Novel Wild Tea Plant Resource with Naturally Low Caffeine and High Theobromine from Guangxi Province, China

Qianting Ma [1, 2, 3, #], Zhongjun Yan [3, #], Xiaolu Yang [4], Aixiang Hou [4], Zhen Liu [3], Shuang Gan [2], Yihuan Yang [2], Yaojin Chen [2], Ruijin Qiu [2, *], Wenliang Wu [1, 3, **]

[1] Longping Agricultural College, Hunan University, Changsha, Hunan 410082, China

[2] Key Laboratory of Liupao Tea Biology and Resource Utilization, Wuzhou Institute of Agriculture Sciences, Wuzhou, Guangxi 543003, China

[3] Hunan Institute of Tea Research, Hunan Academy of Agricultural Sciences, Changsha, Hunan 410125, China

[4] College of Food Science and Technology, Hunan Agricultural University, Changsha, Hunan 410128, China

# These authors contributed equally to this work

* Corresponding author: Email: qiuruijin0925@163.com

** Corresponding author: Email: wwlvip8@163.com

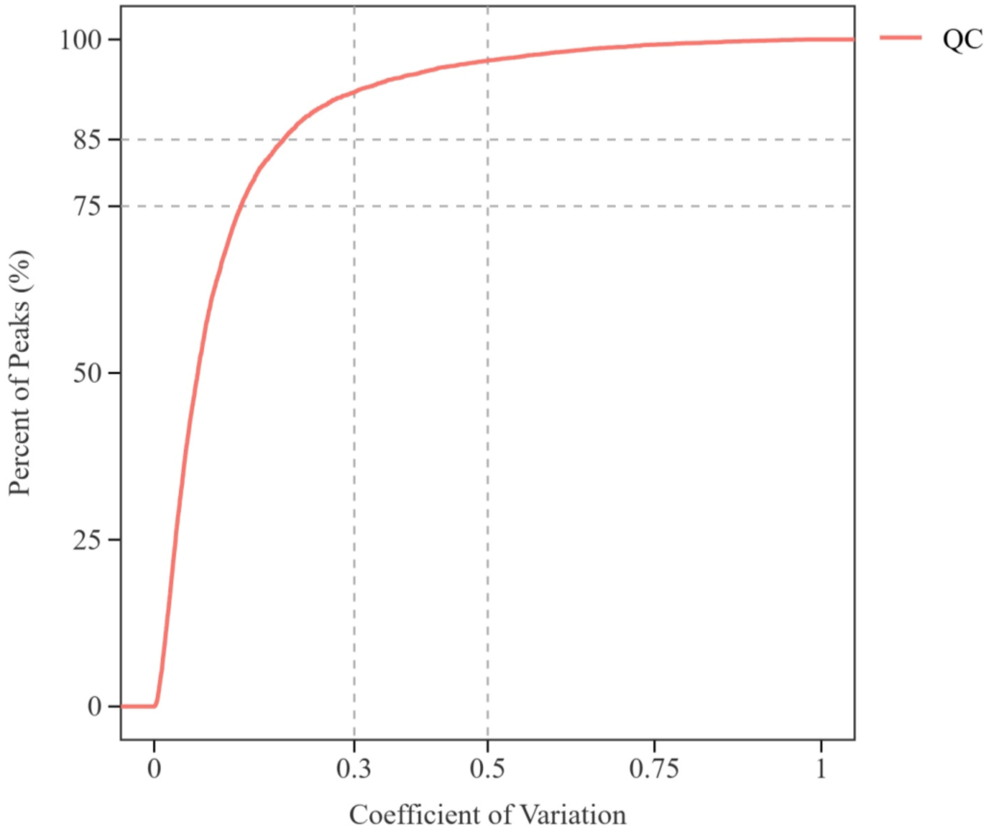

Figure S1. Empirical cumulative distribution function (ECDF) plot of the coefficient of variation (CV) for quality control (QC) samples in non-volatile metabolite analysis.

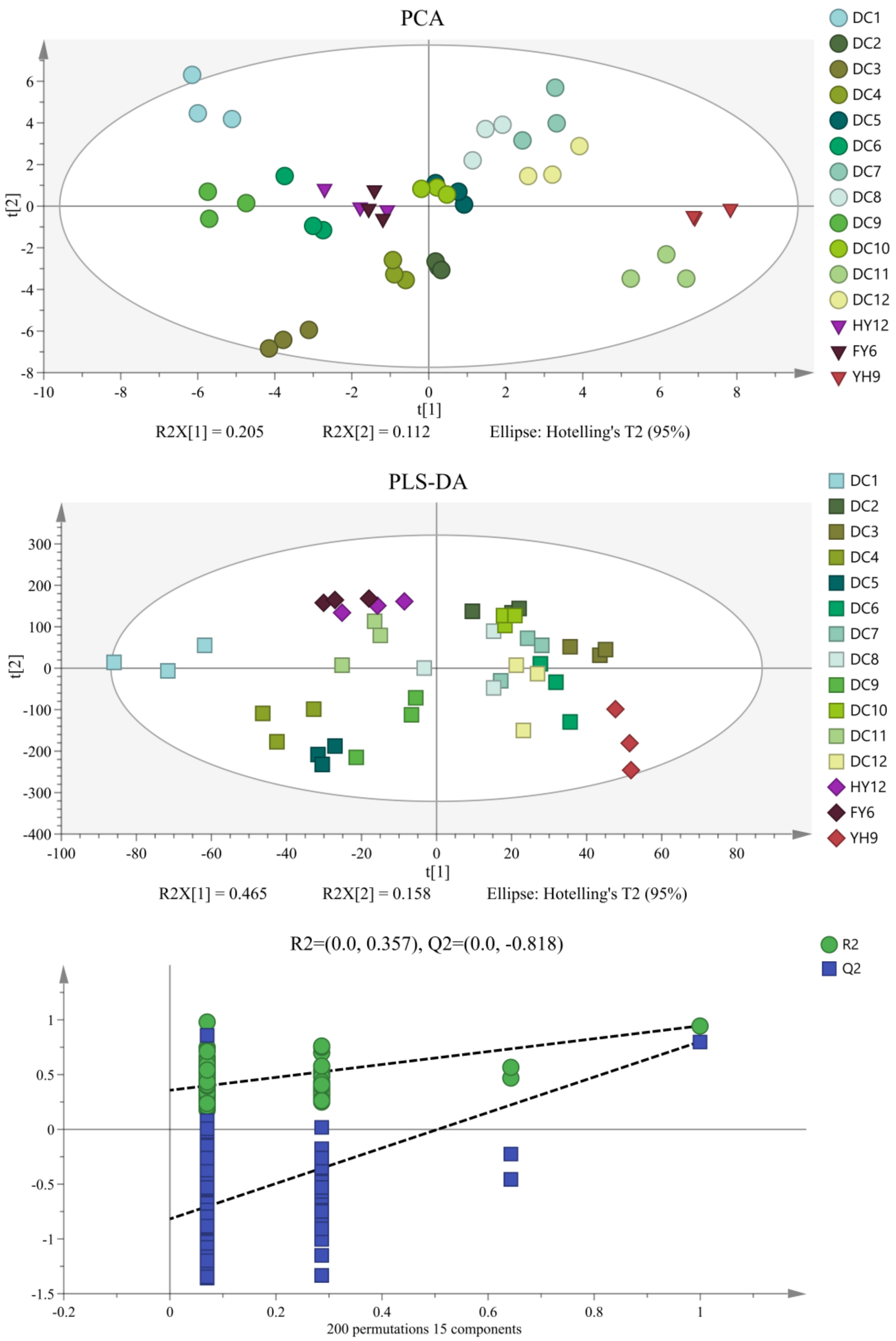

Figure S2. Multivariate statistical analysis of volatile metabolites in ZYC individual plants (DC1-DC12) and CK cultivars (HY12, FY6, and YH9). From top to bottom: PCA score plot, PLS-DA score plot, cross-validation plot of PLS-DA model with 200 permutation tests (intercept: R^2= 0.357, Q^2 = –0.818). ZYC, Zhuyecha (twelve individual plants: DC1-DC12); CK, control (three tea cultivars: HY12, 'Hongyan 12'; FY6, 'Fuyun 6'; YH9, 'Yinghong 9').
